# Surveillance and Vaccine Effectiveness of an Influenza Epidemic Predominated by Vaccine-Mismatched Influenza B/Yamagata-Lineage Viruses in Taiwan, 2011−12 Season

**DOI:** 10.1371/journal.pone.0058222

**Published:** 2013-03-05

**Authors:** Yi-Chun Lo, Jen-Hsiang Chuang, Hung-Wei Kuo, Wan-Ting Huang, Yu-Fen Hsu, Ming-Tsan Liu, Chang-Hsun Chen, Hui-Hsun Huang, Chi-Hsi Chang, Jih-Haw Chou, Feng-Yee Chang, Tzou-Yien Lin, Wen-Ta Chiu

**Affiliations:** 1 Centers for Disease Control, Taipei, Taiwan, Republic of China; 2 Department of Internal Medicine and Graduate Institute of Medical Sciences, National Defense Medical Center, Taipei, Taiwan, Republic of China; 3 Department of Health, Taipei, Taiwan, Republic of China; Melbourne School of Population Health, Australia

## Abstract

**Introduction:**

The 2011−12 trivalent influenza vaccine contains a strain of influenza B/Victoria-lineage viruses. Despite free provision of influenza vaccine among target populations, an epidemic predominated by influenza B/Yamagata-lineage viruses occurred during the 2011−12 season in Taiwan. We characterized this vaccine-mismatched epidemic and estimated influenza vaccine effectiveness (VE).

**Methods:**

Influenza activity was monitored through sentinel viral surveillance, emergency department (ED) and outpatient influenza-like illness (ILI) syndromic surveillance, and case-based surveillance of influenza with complications and deaths. VE against laboratory-confirmed influenza was evaluated through a case-control study on ILI patients enrolled into sentinel viral surveillance. Logistic regression was used to estimate VE adjusted for confounding factors.

**Results:**

During July 2011−June 2012, influenza B accounted for 2,382 (72.5%) of 3,285 influenza-positive respiratory specimens. Of 329 influenza B viral isolates with antigen characterization, 287 (87.2%) were B/Yamagata-lineage viruses. Proportions of ED and outpatient visits being ILI-related increased from November 2011 to January 2012. Of 1,704 confirmed cases of influenza with complications, including 154 (9.0%) deaths, influenza B accounted for 1,034 (60.7%) of the confirmed cases and 103 (66.9%) of the deaths. Reporting rates of confirmed influenza with complications and deaths were 73.5 and 6.6 per 1,000,000, respectively, highest among those aged ≥65 years, 50−64 years, 3−6 years, and 0−2 years. Adjusted VE was −31% (95% CI: −80, 4) against all influenza, 54% (95% CI: 3, 78) against influenza A, and −66% (95% CI: −132, −18) against influenza B.

**Conclusions:**

This influenza epidemic in Taiwan was predominated by B/Yamagata-lineage viruses unprotected by the 2011−12 trivalent vaccine. The morbidity and mortality of this vaccine-mismatched epidemic warrants careful consideration of introducing a quadrivalent influenza vaccine that includes strains of both B lineages.

## Introduction

The influenza B viruses comprise two antigenically distinct lineages, the Yamagata and Victoria lineages, which co-circulate among humans [1]. During influenza seasons a predominant influenza A strain is often present and one of influenza B lineages commonly predominates over the other [2]. Immunization against one influenza B lineage is expected to provide low cross protection against infection with the other lineage [3], [4].

Annual trivalent influenza vaccines contain three vaccine strains, each representing A (H1N1), A (H3N2), and one of the two influenza B lineages, to protect against influenza viruses that are expected to circulate in the upcoming influenza season. The strains are selected for the annual vaccine based on viral surveillance data. However, in some seasons, the vaccine strains did not match the predominant circulating influenza A or B viruses [5]. In Taiwan, the vaccine match rates during July 1987−June 2004 for influenza A (H1N1), A (H3N2), and B viruses were estimated to be 82%, 53%, and 47%, respectively [6]. During July 1999−June 2011 in Taiwan, influenza B predominated over influenza A in three of 12 influenza seasons, including the 2000−01 (B/Yamagata), 2004−05 (B/Victoria and B/Yamagata co-circulating), and 2006−07 (B/Victoria) seasons. Of these, vaccine mismatches of influenza B occurred during the 2004−05 and 2006−07 seasons [7]–[11]. Such mismatches could reduce overall effectiveness and public health benefits of trivalent influenza vaccines [12].

The composition of the 2011−12 northern hemisphere influenza vaccines recommended by the World Health Organization included a B/Brisbane/60/2008-like virus from the B/Victoria lineage [13]. In Taiwan, annual trivalent influenza vaccines have been offered free of charge since 1998 by Taiwan Centers for Disease Control (TCDC) to target populations at increased risk for influenza complications. Among the 23.2 million population in Taiwan, target populations for the 2011–12 season included the elderly (aged ≥65 years), children from the age of 6 months through fourth grade, health care workers, swine and poultry workers, and patients with catastrophic illnesses as defined by the Bureau of National Health Insurance [14]. During October 1–December 31, 2011, 2.67 million doses of free nonadjuvanted inactivated influenza vaccines were available for use among the estimated 4.79 million target populations. By December 31, a total of 2.52 million (94.4%) doses were administered. The overall coverage rate of influenza vaccination among target populations increased from 43.3% during the 2010–11 season to 47.9% during the 2011–12 season in which the estimated coverage rates were 41.2% for adults aged ≥65 years, 40.2% for children aged 0.5–2 years, 26.0% for preschool children ≥3 years, 72.2% for children from first through fourth grade, 85.0% for health care workers, and 57.2% for swine and poultry workers (TCDC, unpublished data, 2012). However, the 2011−12 seasonal influenza epidemic in Taiwan was predominated by the influenza B/Yamagata-lineage viruses that did not match the 2011−12 trivalent vaccine strain. We used the national surveillance data to characterize this vaccine-mismatched epidemic and estimated influenza vaccine effectiveness (VE) during the 2011−12 season.

## Methods

The National Influenza Surveillance System (NISS) in Taiwan consists of sentinel viral surveillance, emergency department (ED) and outpatient influenza-like illness (ILI) syndromic surveillance, and case-based surveillance of influenza with complications and deaths [10]. ILI was defined as acute onset of fever (≥38°C) with respiratory tract symptoms and either myalgia, headache, or extreme fatigue.

### Sentinel Viral Surveillance

Viral surveillance was conducted through approximately 260 sentinel physicians at 160 health care facilities (36 [23%] pediatric-specific) in 21 of the 22 counties or cities in Taiwan. Each sentinel physician was requested to randomly collect throat or nasal swabs from 2–5 ILI outpatients weekly and submitted the swabs to one of the eight collaborating laboratories for influenza testing using viral culture and/or real-time polymerase chain reaction (RT-PCR) [7]. A subset of the influenza viruses was randomly selected and submitted to TCDC for gene sequencing, oseltamivir resistance testing and antigenic characterization. Methods of influenza RT-PCR and viral culture have been described [6]. Antigenic characterization was determined using the hemagglutination inhibition (HI) assays with ferret antisera [15]. Oseltamivir-resistant influenza viruses were detected by using DNA sequences and 50% inhibitory concentration analysis of neuraminidase activity [16].

### ED and Outpatient ILI Syndromic Surveillance

TCDC conducts surveillance of ILI-related emergency department (ED) visits through the Real-time Outbreak and Disease Surveillance (RODS) system [17]. RODS consists of 170 hospitals throughout Taiwan with a coverage of approximately 85% of all ED visits in Taiwan. RODS collected and analyzed the *International Classification of Diseases, Ninth Revision, Clinical Modification* (ICD-9-CM) codes of all ED visits at participating hospitals on a daily basis. ILI-related ED visits were detected by using the pre-defined ICD-9-CM diagnostic codes for respiratory syndrome of RODS [18]. Information on ILI-related outpatient visits is collected through the computerized claim database of the National Health Insurance (NHI) system, a single-payer compulsory social insurance plan which centralizes reimbursement of health care funds and covers >99% of population in Taiwan. ILI-related outpatient visits were detected by using the ICD-9-CM codes for influenza and pneumonia (480−487). NHI submitted aggregated data on ILI-related outpatient visits by age groups and geographic areas to TCDC daily for analysis.

### Case-based Surveillance of Influenza with Complications and Deaths

Influenza with complications is a nationally notifiable condition in Taiwan. A suspected case was defined as a hospitalization or death ≤4 weeks of ILI onset associated with at least one of the following complications: pulmonary complications, neurological complications, cardiac complications (myocarditis or pericarditis), and invasive bacterial infection. A confirmed case was a suspected case with a positive influenza RT-PCR and/or viral culture at collaborating laboratories. Physicians are required to report suspected cases to corresponding local health departments ≤7 days of case identification and submit respiratory specimens (preferentially throat swabs) in all suspected cases to collaborating laboratories that participate in influenza viral surveillance. Local public health professionals verified case characteristics including presenting symptoms, dates of illness onset and hospitalizations, types of complications, vaccination status, and underlying medical conditions based on physician’s reports and hospital records. The National Death Registry was linked to identify deaths that had occurred ≤4 weeks of illness onset among confirmed cases.

### Antiviral Stockpile Release

In preparation for pandemic influenza, TCDC has stocked approximately 5.75 million courses of antiviral medications including oseltamivir, zanamivir, and peramivir to cover 25% of the population. A committee consisting of virologists, infectious disease specialists, and public health professionals advised TCDC on the policy of antiviral stockpile release based on magnitude of the epidemic, amount and expiration dates of stockpiled antivirals, and prevalence of drug resistance among circulating strains. During the 2011−12 season, pandemic influenza antiviral stockpile was continually released for free medical use with the following indications: (1) suspected or confirmed cases of influenza with complications; (2) ILI with signs and symptoms of progressive disease [19]; (3) ILI in pregnancy; (4) ILI in patients with chronic diseases of heart, lungs, liver, or kidneys, diabetes mellitus, or other critical medical conditions; (5) ILI in patients with a body mass index ≥35 kg/m^2^; (6) Influenza outbreaks. In response to the vaccine-mismatched influenza B epidemic, TCDC added two indications for free antiviral release during December 1, 2011−March 31, 2012: (1) ILI with fever for ≥48 hours; (2) ILI in patients who had been in close contact with other ILI patients at home, school, or workplace.

### Surveillance Data Analysis

We included surveillance data and cases reported during the TCDC-defined 2011−12 influenza season (July 1, 2011−June 30, 2012) which started in week 26, 2011 and ended in week 26, 2012. The Taiwan Census data as of June 2011 available from the Department of Household Registration were used as denominators to calculate overall and age-specific reporting rates of influenza with complications and influenza-associated mortality rates. Descriptive statistics were calculated and figures were produced by using Microsoft Excel (2007). Bivariate analyses were conducted using Wilcoxin two-sample test for continuous variables and chi-square or Fisher’s exact test for categorical variables.

### VE Study

Effectiveness of the 2011−12 trivalent vaccine against laboratory-confirmed influenza in Taiwan was evaluated through a case-control study using the sentinel viral surveillance data. From November 1, 2011 through April 30, 2012, each sentinel physician was requested to randomly select up to five ILI outpatients aged ≥6 months weekly with nasal/throat swabs and information of demographics, vaccination status and comorbidity collected. The enrollment period ended on April 30, 2012 to provide in-season VE estimates. Patients were included into this VE analysis if results of influenza testing on nasal/throat swabs and information of vaccination status were both available. We defined those with a positive influenza culture and/or RT-PCR as case-patients and those with negative influenza test results as controls. A valid vaccination was defined as >14 days between receiving a dose of the 2011−12 influenza vaccine and symptom onset. VE was calculated as (1 - odds ratio [OR] for laboratory-confirmed influenza among vaccinated versus unvaccinated patients) x 100%. Logistic regression was used to estimate adjusted VE and 95% confidence intervals (CI) using OR adjusted for age (<9, 9−49, or >49 years), residing regions (northern or southern Taiwan), underlying medical conditions (presence or not), and intervals between symptom onset and specimen collection (0−2 or >2 days). Variables were analyzed using SAS version 9.2® (SAS Institute Incorporated, Cary, North Carolina, USA).

### Ethics Statement

Data obtained for this study was for surveillance purposes. TCDC determined this analysis a public health response and exempt from Institutional Review Board review.

## Results

### Sentinel Viral Surveillance

**Figure 1:**
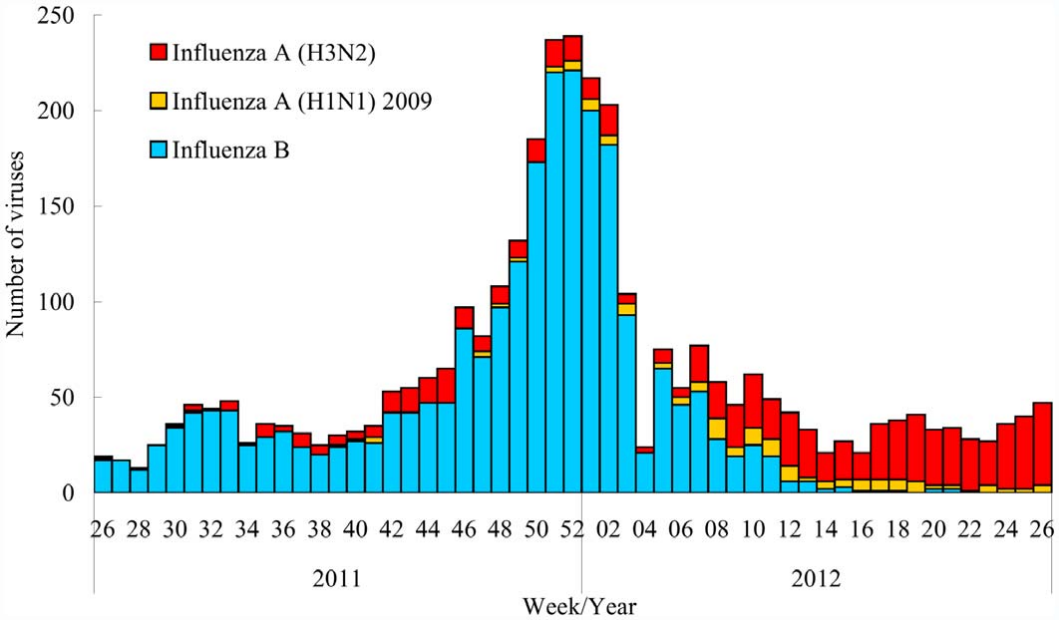
Number of influenza-positive specimens by virus types and subtypes tested at collaborating laboratories in Taiwan, 2011−2012 season.

During the 2011−2012 season, 3,285 (22.0%) of 14,943 respiratory specimens tested positive for influenza. Among the influenza-positive specimens, 2,382 (72.5%) were influenza B, 757 (23.0%) were influenza A (H3N2), and 146 (4.4%) were influenza A (H1N1) 2009. Influenza B was the predominantly circulating type during July 2011−February 2012 (week 26, 2011−week 9, 2012) whereas influenza A (H3N2) predominated during March to June 2012 (week 10, 2011−week 26, 2012) (Fig. 1).

Of 329 influenza B viral isolates tested during the 2011−2012 season, 287 (87.2%) belonged to the Yamagata lineage and were antigenically similar to the B/Florida/4/2006-like strain contained in the 2008−09 influenza trivalent vaccine. All of the other 42 influenza B/Victoria-lineage viruses were antigenically similar to the B/Brisbane/60/2008-like strain contained in the 2011−12 influenza trivalent vaccine. Oseltamivir resistance testing was conducted among 293 influenza B, 213 influenza A (H3N2), and 52 influenza A (H1N1) 2009 viruses during the 2011−2012 season. No mutation associated with oseltamivir resistance was detected.

### ILI Syndromic Surveillance

**Figure 2:**
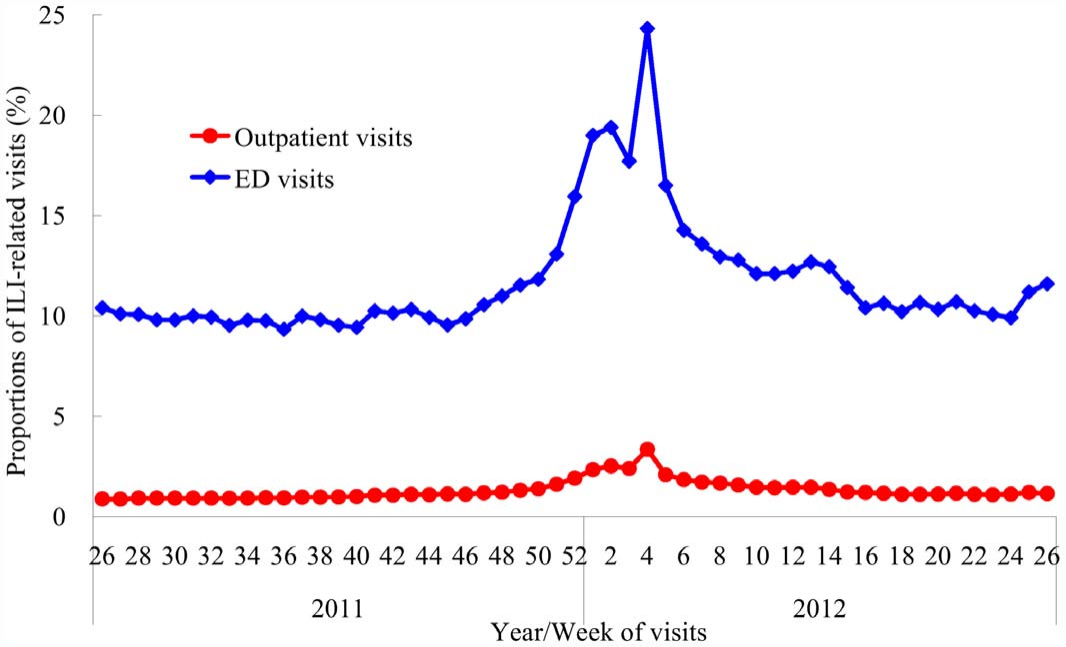
Proportion of outpatient visits and emergency department (ED) visits being influenza-like illness (ILI)-related in Taiwan, 2011−2012 season.

The proportions of ILI-related visits among all NHI-covered outpatient visits and all RODS-enrolled ED visits had both steadily increased since week 46, 2011 and reached the first peaks at week 2, 2012 (Fig. 2). A bigger increase occurred during the 2012 Lunar New Year holidays (week 4) when ILI accounted for 3.35% and 24.32% of all outpatient visits and ED visits, respectively. A smaller increase occurred during week 24−26, 2012.

### Case-based Surveillance of Influenza with Complications and Deaths

**Figure 3:**
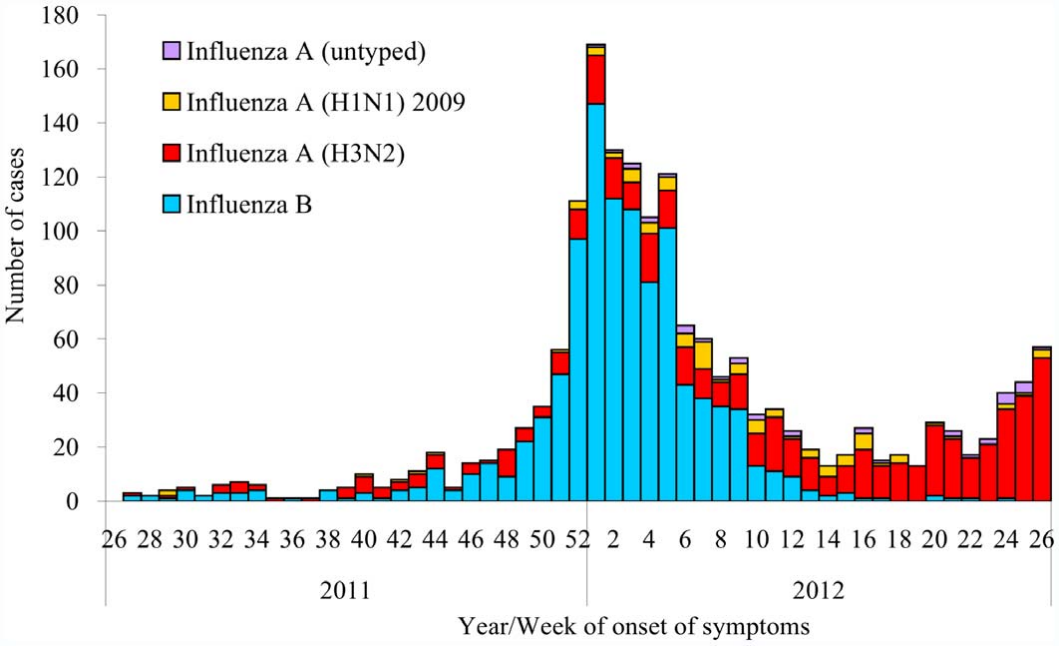
Number of confirmed cases of influenza with complications by virus types and subtypes in Taiwan, 2011−2012 season.

During the 2011−2012 season, TCDC were notified of 2,675 suspected cases of influenza with complications. Of them, 1,704 were confirmed cases, including 154 (9.0%) deaths. The number of confirmed cases, predominated by influenza B, had steadily increased since week 46, 2011 and reached the peak at week 1, 2012 (Fig. 3). Influenza A (H3N2) had predominated over influenza B since week 11 and steadily increased since week 22, 2012. Overall influenza B accounted for 1,034 (60.7%) of the confirmed cases and 103 (66.9%) of the deaths. In comparison, influenza A (H3N2) accounted for 548 (32.2%) of the confirmed cases and 41 (26.6%) of the deaths; influenza A (H1N1) 2009 accounted for 87 (5.1%) of the confirmed cases and 8 (5.2%) of the deaths; influenza A (untyped) accounted for 35 (2.1%) of the confirmed cases and 2 (1.3%) of the deaths. Case-fatality rates of influenza B, A (H3N2), A (H1N1) 2009, and A (untyped) were 10.0%, 7.5%, 9.2%, and 5.7%, respectively (p = 0.37).

During the 2011−12 season, the overall reporting rates of confirmed influenza with complications and deaths were 73.5 and 6.6 per 1,000,000 people, respectively, and were the highest among the age groups of ≥65 years, 50−64 years, 3−6 years, and 0−2 years, respectively (Table 1). The median ages of confirmed cases of influenza B, A (H3N2), A (H1N1) 2009, and A (untyped) were 58, 70, 49, and 60 years, respectively (p<0.0001). Underlying medical conditions were present in 1,054 (61.9%) of the 1,704 confirmed cases. The most common underlying medical conditions among confirmed cases were metabolic diseases (n = 355, 33.7%), cardiovascular diseases (n = 284, 26.9%), chronic pulmonary diseases (n = 256, 24.2%), and chronic kidney diseases (n = 121, 7.1%). Case-fatality rates were higher among cases with underlying medical conditions than those without underlying medical conditions (12.4% vs. 3.5%, p<0.0001).

**Table 1:** Number and reporting rate of laboratory-confirmed complicated influenza and influenza-associated deaths by age groups in Taiwan, 2011−2012 season.

Age group (years)	B cases (deaths)	A(H3N2) cases (deaths)	A(H1N1) cases (deaths)	A(untyped) cases (deaths)	All cases (deaths)	Age-specific reporting rate (mortality rate)*
0−2	22 (2)	13 (1)	2 (0)	0 (0)	37 (3)	67.4 (5.5)
3−6	50 (5)	18 (1)	3 (1)	2 (0)	73 (7)	87.8 (8.4)
7−18	72 (4)	14 (2)	2 (0)	0 (0)	88 (6)	25.3 (1.7)
19−24	12 (0)	4 (0)	10 (0)	0 (0)	26 (0)	13.6 (0)
25−49	220 (7)	70 (3)	27 (1)	10 (0)	327 (11)	34.7 (1.2)
50−64	273 (28)	122 (8)	18 (3)	9 (1)	422 (40)	94.1 (8.9)
≥65	385 (57)	307 (26)	25 (3)	14 (1)	731 (87)	293.2 (34.9)
Total	1034 (103)	548 (41)	87 (8)	35 (2)	1704 (154)	73.5 (6.6)

* Age-specific reporting rates and mortality rates were per 1,000,000 people.

Administration of the 2011−12 trivalent influenza vaccine before illness was reported in 173 (10.2%) of the 1,704 confirmed cases, including 19 (12.3%) of the 154 deaths. Vaccination rates among confirmed cases of influenza B, A (H3N2), A (H1N1) 2009, and A (untyped) were 10.2%, 10.9%, 6.9%, and 5.7%, respectively (p = 0.54).

### Antiviral Stockpile Release

During the 2011−12 season, 387,608 courses of stockpiled antiviral agents were released and prescribed, comprising 6.7% of TCDC’s pandemic influenza antiviral stockpile. Of them, 370,297 (95.5%) courses were prescribed under the two newly added indications during December 2011−March 2012, including 361,037 courses of oseltamivir, 6,779 courses of zanamivir, and 153 courses of peramivir.

### VE Study

During the 6-month study period, nasal/throat swabs and information of vaccination status were collected in 918 patients. Seven patients were excluded because of age <6 months (5 patients) and mislabeled specimens (2 patients). Of 911 patients included for the VE study, 602 (66.1%) were aged <9 years and 259 (28.4%) were aged 9−49 years, and 50 (5.5%) were aged >49 years. A total of 495 (54.3%) resided in northern Taiwan. Underlying medical conditions were present in 39 (4.3%) patients. Intervals between symptom onset and specimen collection were 0−2 days in 558 (83.2%) patients. Influenza testing was positive in 296 (32.5%) patients (case-patients) and negative in 615 (67.5%) patients (controls). Case-patients and controls differed significantly in age group distribution and residing regions, but did not differ significantly in sex, presence of underlying medical conditions, and intervals between symptom onset and specimen collection (Table 2).

**Table 2:** Characteristics of cases and controls in the vaccine effectiveness study.

Characteristics	Cases (N = 296)	Controls (N = 615)	*P*
	n (%)	n (%)	
Age group (years)			0.0002
0.5−8	169 (57)	433 (70)	
9−49	110 (37)	149 (24)	
≥50	17 (6)	33 (5)	
Sex			0.86
Female	138 (47)	283 (46)	
Male	158 (53)	332 (54)	
Residing region			<0.0001
Northern Taiwan	131 (44)	364 (59)	
Central and southern Taiwan	165 (56)	251 (41)	
Underlying medical conditions			0.25
Yes	16 (5)	23 (4)	
No	280 (95)	592 (96)	
Interval between symptom onset and specimen collection (days)			0.53
0−2	243 (82)	515 (84)	
≥3	53 (18)	100 (16)	

Vaccinated patients accounted for 9 (18.4%) of 49 influenza A-positive cases, 87 (35.2%) of 247 influenza B-positive cases, and 169 (27.5%) of 615 controls. Crude VE was −27% (95% CI: −71, 6) against all influenza, 47% (95% CI: −11, 75) against influenza A, and −49% (95% CI: −103, −9) against influenza B. In logistic regression, adjusted VE was −31% (95% CI: −80, 4) against all influenza, 54% (95% CI: 3, 78) against influenza A, and −66% (95% CI: −132, −18) against influenza B.

## Discussion

The 2011−12 seasonal influenza epidemic in Taiwan was predominantly caused by the B/Florida/4/2006-like strain from the B/Yamagata lineage. Over the past decade, the largest seasonal influenza epidemic in Taiwan occurred during the 2010−2011 season and was predominated by influenza A (H3N2) and A (H1N1) 2009 viruses, resulting in 1,785 confirmed cases of influenza with complications, including 181 (10.1%) deaths [10]. The 2011−12 influenza epidemic had a similar number of cases and deaths, indicating that influenza B could result in an epidemic with substantial morbidity and mortality.

The VE study results suggested the 2011−12 trivalent influenza vaccine moderately protected against influenza A, consistent with the VE estimates of 43−54% against medically attended influenza A (H3N2) reported in two European studies in the context of A (H3N2) antigenic variation from the vaccine strain [20],[21]. However, our results demonstrated an unexpected harmful effect from the vaccine with respect to influenza B. An explanation was unmeasured confounding such as healthy vaccinee effect and social determinants [22]. However, these confounding would have erroneously reduced VE estimates against influenza A as well, which did not seem to have occurred in our study. During the influenza A (H1N1) 2009 pandemic, several studies demonstrated increased risk of A (H1N1) 2009 illness among recipients of the 2008−09 trivalent vaccine [23]–[27]. Hypotheses of possible biologic mechanisms included cross-protection block, antibody-dependent enhancement, and temporary immunity [24], [28]–[29]. Trivalent influenza vaccine recipients were reportedly at increased risk of non-influenza respiratory virus infection from lack of temporary non-specific immunity following influenza virus infection [30]. A recent study based on animal models and virus neutralization assays in children concluded that seasonal influenza vaccination was associated with reduced virus-specific CD8+ T cell response and impaired induction of heterosubtypic immunity against influenza A [31]. Interference of trivalent vaccines on cross-lineage immunity against influenza B has not been reported. Further studies are needed to determine whether trivalent influenza vaccine recipients are less likely than non-recipients to develop cross-lineage immunity against strains of a vaccine-unmatched influenza B lineage.

The 2009−10 and 2010−11 seasons in Taiwan was predominated by vaccine-matched influenza A viruses, leading the public to confide in the effectiveness and benefits of influenza vaccination [10]. Predominance of B/Yamagata-lineage viruses was not evident until July 2011 when production of the 2011−12 trivalent vaccine was already in progress. This epidemic underscores the limitation of annual trivalent vaccines in containing only one influenza B strain that might not match the predominant B lineage in the upcoming season. In Taiwan, vaccine mismatches of influenza B had occurred in five of 10 influenza seasons during July 2002−June 2012 [6]–[11]. Such mismatches are common and could likely lead to reduced willingness of the public towards vaccination against seasonal influenza. A quadrivalent influenza vaccine that contains viruses from both circulating B lineages is a proposed solution and could reduce influenza-related morbidities and mortalities if vaccine production capacity is not adversely affected [32]–[34]. However, a quadrivalent vaccine is likely associated with increased cost, resulting in additional financial burden to the government-funded free influenza vaccination program in Taiwan. Inclusion of an additional strain might prolong the vaccine production process. Earlier selection of candidate strains would facilitate timely production but might increase the likelihood of vaccine mismatches. These issues should be considered before adaptation of a quadrivalent influenza vaccine into the vaccination program.

During this vaccine-mismatched epidemic, release of pandemic antiviral stockpile was used as a control measure to reduce viral transmission. Although all of the circulating influenza viruses sampled during the 2011−12 season remained susceptible, acquired antiviral resistance was not optimally assessed because the NISS did not target populations at increased risk for antiviral resistance such as those who had received antiviral treatment or were immunocompromised. Epidemic-driven release of pandemic antiviral stockpile should be balanced with stockpile adequacy, cost associated with antiviral procurement and distribution, risk of emergence of oseltamivir resistance, and reportedly low sensitivity of influenza B viruses to oseltamivir [35]–[38].

This study is subject to the following limitations. First, the number of influenza with complications and deaths might have been underestimated because of underdiagnoses and underreporting. However, underreporting was likely uncommon because a patient would be eligible for free antivirals once reported as a suspected or confirmed case. Second, the dramatic increase in the proportions of ILI-related ED and outpatient visits during the 2012 Lunar New Year Holidays (week 4) reflected change in availability of clinical services and were not necessarily indicative of an increased frequency of ILIs in the community. Because routine outpatient services are generally closed during Lunar New Year Holidays, patients tend to seek medical help only when they experience acute illnesses, including ILIs, and have an increased likelihood to present to EDs. The first deflection point (week 2) might rather represent the true peak of this epidemic, which also approximated the peaks detected by sentinel viral surveillance (week 52) and case-based surveillance (week 1). Third, the VE study sample underrepresented elderly patients and might overrepresent those with increased access to medical services or care-seeking behaviors (healthy vaccinee effect). The sample was also too small for age- or target population-stratified estimates. Finally, we only evaluated VE against laboratory-confirmed influenza in outpatient settings; other outcomes (for example, hospitalization) were not assessed.

In conclusion, the 2011−12 seasonal influenza epidemic in Taiwan was predominated by the influenza B/Yamagata-lineage viruses that were not protected by the 2011−12 trivalent vaccine. The morbidity and mortality of this influenza epidemic call for the need of careful consideration of a quadrivalent influenza vaccine that includes viruses of both circulating B lineages to prevent vaccine mismatches.
